# Successful Reversal of Acute Kidney Failure by Ultrasound-Accelerated Thrombolysis of an Occluded Renal Artery

**DOI:** 10.1155/2014/205646

**Published:** 2014-09-08

**Authors:** Renske Konings, Rutger J. Lely, Shaikh A. Nurmohamed, Arjan W. J. Hoksbergen

**Affiliations:** ^1^Department of Surgery, VU University Medical Center, P.O. Box 7057, 1007 MB Amsterdam, The Netherlands; ^2^Department of Interventional Radiology, VU University Medical Center, Amsterdam, The Netherlands; ^3^Department of Nephrology, VU University Medical Center, Amsterdam, The Netherlands

## Abstract

*Purpose*. To describe the treatment of renal artery thrombosis with ultrasound-accelerated thrombolysis and discuss the management of prolonged renal ischemia. *Case*. A 76-year-old patient with a single functional kidney, mild chronic renal impairment, and a recent history of endovascular repair of a thoracoabdominal aneurysm with an aortic branch graft presented with acute flank pain, anuria, and renal failure. The side branch from the aortic stent graft to his single, right, functional kidney appeared to be completely thrombosed. Symptoms had started after cessation of oral anticoagulants because of a planned mastectomy for breast cancer. After identification of the occlusion, ultrasound-accelerated thrombolysis was started 19 hours after the onset of anuria. Angiography, 4 hours after beginning of therapy, already showed partial dissolution of the thrombus and angiographic control after 18 hours showed complete patency of the renal artery side branch. Despite a long period of ischemia, renal function was completely recovered. *Conclusion*. In patients with acute renal ischemia due to thrombosis of the renal artery, complete recovery of function can be achieved with ultrasound-accelerated thrombolysis, even after prolonged periods of ischemia.

## 1. Introduction

Acute occlusion of the renal artery requires instant diagnosis and revascularisation because prolonged ischemia can cause irreversible renal dysfunction [1, 2]. A number of reports have tried to identify patients who could benefit from revascularisation [3–11]. Several cases have been published describing successful revascularisation by endovascular therapy [1, 3, 7, 10, 11].

To our knowledge, this is the first case describing the use of catheter-directed ultrasound-accelerated thrombolysis for treatment of acute renal failure caused by thrombosis of a renal artery. The clinical course will be presented and the indication for revascularisation after prolonged periods of renal ischemia will be discussed.

## 2. Case Report

A 76-year-old man with a medical history of hypertension, atrial fibrillation, type 2 diabetes mellitus, and mild chronic renal impairment with a single functional kidney was referred to our hospital because of a Crawford type 3 thoracoabdominal aneurysm with a diameter of 6.4 cm. There was a preexisting occlusion of the left renal artery. Baseline serum creatinine was 1.81 mg/dL. The estimated glomerular filtration rate (eGFR) using the modified diet and renal disease study equation (MDRD) was 37 mL/min/1.73 m^2^.

**Figure 1:**
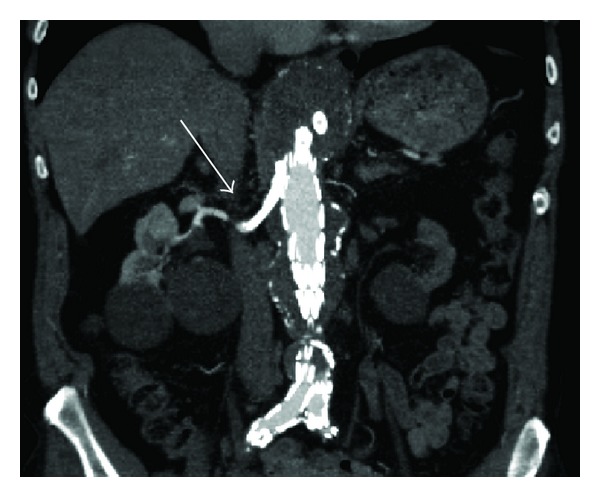
Postoperative CTA showing adequate position of the aortic branch graft with patent side branch in the right renal artery (arrow).

**Figure 2:**
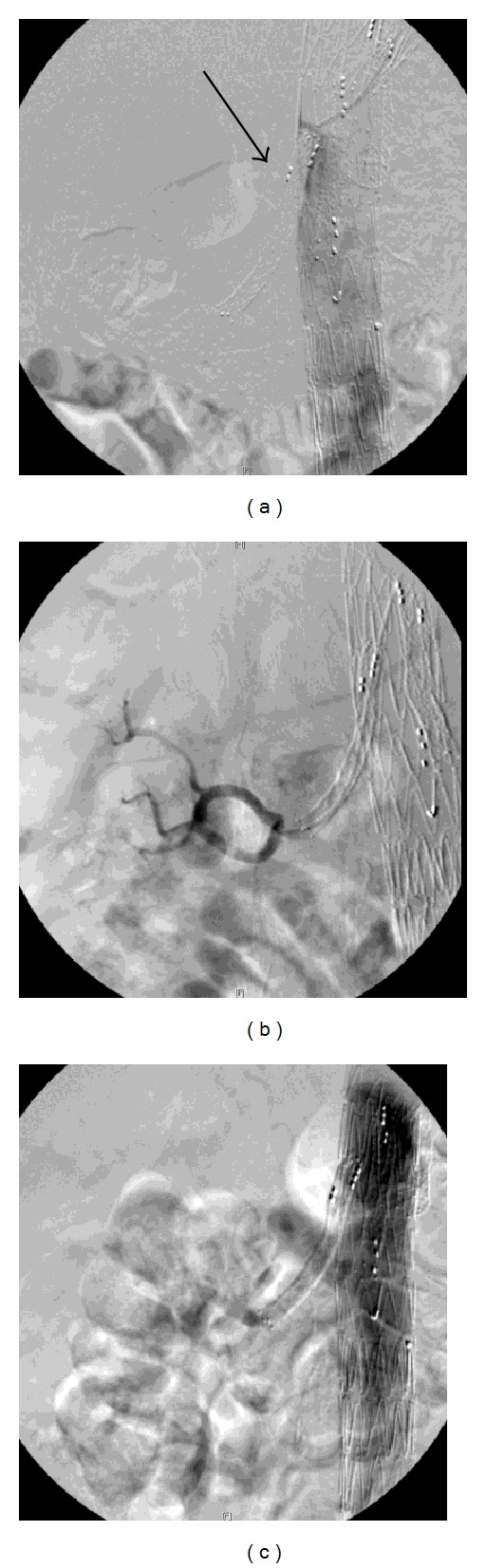
(a) Angiography (via left brachial access) just proximal of the right renal branch of the stent graft showing occlusion of the side branch in the renal artery (arrow). No opacification of the right kidney is identified, consistent with absent perfusion to the right kidney. (b) EKOS thrombolysis catheter with a working length of 6 cm (EKOS Endowave system; EKOS Corporation, Bothell, WA, USA) placed through side branch into right renal artery with filling of segmental renal artery branches after contrast injection. (c) Patent side branch after successful thrombolysis.

Endovascular repair was performed with an aortic branch graft including side branches for the celiac trunk, superior mesenteric artery (SMA), and the right renal artery. Covered stents were placed through the side branches in the target vessels and these were relined with nitinol self-expanding stents. The procedure was complicated by diplopia caused by occipital infarction, despite intraoperative heparinisation and immediate postoperative readministration of anticoagulant therapy (coumarin and clopidogrel). Postoperative serum creatinine remained unchanged. Three weeks after surgery, clopidogrel was discontinued because of side effects. Follow-up CT-angiography 6 weeks after discharge showed adequate position of the aortic branch graft (Figure 1), patency of the visceral side branches, and target vessels without kinking of the (covered) stents. However, a mass in the left mamma was found. Additional diagnostic tests showed an invasive ductal carcinoma. Subsequently the patient was planned for mastectomy with sentinel node procedure. Anticoagulant therapy was discontinued 3 days before surgery. Upon admission for the planned surgery, the patient reported anuria for approximately 12 hours and right flank pain that had started early that morning. Blood test showed an increase in serum creatinine to 5.37 mg/dL. Ultrasound investigation showed an occlusion of the stents in the right renal artery side branch and minimal venous flow suggesting acute ischemic kidney injury. The side branches to the celiac trunk and SMA were patent without presence of thrombus. Systemic heparinisation was started immediately and the mastectomy was cancelled. Angiography was performed via left brachial access and occlusion of the right renal artery side branch was confirmed (Figure 2(a)). Via direct transcatheter injection, a single bolus of 250.000 IE urokinase was administered into the right renal side branch and an EKOS thrombolysis catheter (EKOS Endowave system; EKOS Corporation, Bothell, WA, USA) with a working length of 6 cm was placed in the occluded renal side branch (Figure 2(b)). Intra-arterial infusion with 100.000 IU/h of urokinase was started together with 10.000 IU/24 h of heparin via the side port of the sheath. Thrombolysis was started 19 hours after the beginning of anuria. Angiographic control 4 hours later showed some dissolution of the thrombus and thrombolysis was continued. Approximately 8 hours after the start of thrombolysis, diuresis recovered and right flank pain diminished. Second angiographic control 18 hours after beginning of therapy demonstrated complete lysis of the thrombus with a patent side branch of the aortic stent graft and adequate flow through the renal artery (Figure 2(c)), after which thrombolysis was stopped. Subsequently, anticoagulation with low molecular weight heparin was started. The serum creatinine increased to a maximum of 10.44 mg/dL on day 3 after procedure, requiring dialysis on days 3 and 5. Hereafter, there was partial renal function recovery (eGFR (MDRD) of 10 mL/min/1.73 m^2^), after which mastectomy was performed without cessation of anticoagulant therapy. Postoperative coumarin was started and the patient was discharged on day 9, after an uneventful postoperative course. Renal function continued to improve, and one month after thrombolysis, renal function approached baseline function (creatinine 2.33 mg/dL; eGFR (MDRD) 27 mL/min/1.73 m^2^). One year after surgery, the patient was doing well and renal function was stable and the same as before the aneurysm repair. All side branches of the aortic branch graft were patent and the aneurysm was shrinking.

## 3. Discussion

**Figure 3:**
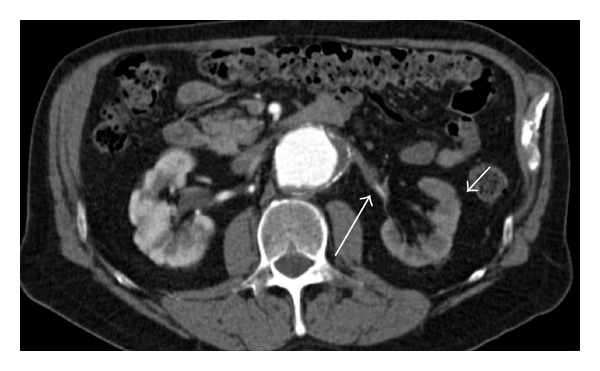
Preoperative CTA showing left kidney with preexistent occluded renal artery and contrast-enhancement in the cortical area (cortical rim sign, small arrow) with distal renal artery perfusion suggesting collateral (perirenal) flow (large arrow).

Symptoms of spontaneous thromboembolic renal artery occlusion might be nonspecific and misleading. In case of typical symptoms such as flank pain and oligoanuria, specifically in patients with a significant vascular history, one should consider this diagnosis [7]. Warm ischemia time tolerance of the kidney is limited to a short time frame. After renal blood supply is interrupted, this period is often presumed to have passed once renal artery occlusion is diagnosed [6]. Since symptoms can be misleading, diagnosis and treatment are often delayed. Once ischemia results in renal infarction, reestablishment of blood flow will not result in improvement of renal function [2]. The period of tissue viability of the kidney is considered to be 1–3 hours [1, 2, 6, 12]. In contrast, there are some reports which suggest that renal function can be regained after longer periods of ischemia. Restoration of renal function has been reported several hours up to several days after initial symptoms [1, 3, 7, 10, 11]. In this case the sudden increase of serum creatinine of more than 3 mg/dL at admission implied an ischemic insult existing for several days, suggesting a gradual thrombosis of the renal side branch of the aortic stent graft. Given the duration of anuria, occlusion due to complete thrombosis of the renal artery side branch was assumed to exist for approximately 19 hours before therapy started. Compromised perfusion of the renal artery may be compensated by collateral blood flow originating from perirenal capsular arteries. This may provide sufficient perfusion pressure to delay irreversible tissue loss after several hours to several days. In our case the presence of collateral circulation is illustrated in the left kidney with the preexistent occluded renal artery, showing a cortical rim sign and contrast-enhancement in the distal renal artery (Figure 3) [12].

In acute kidney failure due to thromboembolic occlusion of a renal artery it is crucial to identify which patients would benefit from renal revascularisation. Multiple variables, including mechanism of occlusion, underlying renal disease, extent and location of thrombus, presence of collateral vessels, and duration of anuria, have been described to predict whether successful restoration of renal function is likely [9, 11, 13]. Anuria time seems to be the best indicator for duration of ischemia and is reversely correlated with recovery of renal function [9].

Considering the aforementioned variables, the ischemia time period after which renal artery revascularisation might still be successful is very difficult to define. The positive result in this case and in some other case reports shows that kidney function might be restored after substantially longer periods of ischemia than the supposed period of 1–3 hours [1, 2, 6, 12].

Selective catheter-directed thrombolysis is minimally invasive, safe, and efficacious in treating acute thromboembolic renal artery occlusion [1, 3, 10, 11]. Based on earlier experience [14] and the recently reviewed literature [15], we found that ultrasound-accelerated thrombolysis provided faster thrombus dissolution than standard thrombolysis. Therefore, this promising new technique might be especially valuable in patients with kidney failure due to thromboembolic renal artery occlusion.

## 4. Conclusion

Urgent revascularisation in acute renal artery occlusion is essential to preserve kidney function, which might be restored after substantially longer periods of ischemia than previously thought. Catheter-directed ultrasound-accelerated thrombolysis is an attractive alternative to standard thrombolysis due to faster dissolution of thrombus. In this case of acute renal artery thrombosis, restoration of diuresis was achieved within 8 hours after beginning of thrombolytic therapy.
